# Impact of hematuria on long term kidney outcomes in adults with immunoglobulin A nephropathy: a systematic literature review and meta-analysis

**DOI:** 10.3389/fneph.2026.1899597

**Published:** 2026-08-12

**Authors:** Joel Iff, Ankit Shah, Rajeshwari Nair, Mirko Fillbrunn, Ogo Egbuna, Manish Maski, Rui Huang, Dumingu Aparna Gomes, Edward Tuttle, Whitney Longstaff, Janice Stricker-Shaver, Beth Barber

**Affiliations:** 1 Vertex Pharmaceuticals Incorporated, Boston, MA, United States; 2 Analysis Group Inc., Boston, MA, United States; 3 Analysis Group Inc., London, United Kingdom; 4 Analysis Group Inc., Menlo Park, CA, United States; 5 Maple Health Group, LLC, New York, NY, United States

**Keywords:** adults, IgA nephropathy, kidney outcomes, persistent microhematuria, review

## Abstract

**Introduction:**

Hematuria is the most common clinical manifestation of Immunoglobulin A nephropathy (IgAN) and has been associated with adverse long-term kidney outcomes, although its prognostic significance has not been clearly established. This systematic literature review and meta-analysis aimed to evaluate whether hematuria is a prognostic marker of long-term kidney outcomes.

**Methods:**

A search of PubMed/MEDLINE and Embase (January 2005 to June 2025) identified studies assessing persistent or single-timepoint measurement of microhematuria or macrohematuria in relation to kidney outcomes. Studies meeting predefined criteria were qualitatively synthesized, and those with comparable outcome definitions were included in meta-analyses using random-effects models.

**Results:**

Forty-three studies were included in the systematic literature review, of which 15 study entries from 13 publications were included in meta-analyses. Across studies, persistent microhematuria was consistently associated with worse long-term kidney outcomes. In pooled analyses, microhematuria (both persistent and single-timepoint measurements) was associated with an increased risk of end-stage kidney disease or related outcomes (hazard ratio [HR] 2.33, 95% confidence interval [CI] 1.94–2.80), with a stronger and consistent association observed for persistent microhematuria (HR 2.57, 95% CI 1.94–3.41). Single-timepoint measurement of microhematuria showed a nonsignificant directional association with substantial variability across studies, whereas macrohematuria (predominantly measured at a single timepoint) was associated with improved kidney outcomes (HR 0.56, 95% CI 0.37–0.83).

**Discussion:**

These findings support persistent microhematuria as a strong and consistent predictor of kidney failure in IgAN, substantiating its role as a clinically relevant prognostic marker.

## Introduction

Immunoglobulin A nephropathy (IgAN) is the most common type of primary glomerulonephritis globally and a progressive immune-mediated kidney disease associated with substantial unmet medical need (1, 2). Its pathogenesis is characterized by mesangial deposition of IgA-containing immune complexes leading to hypercellularity and glomerular inflammation, manifesting clinically as hematuria and proteinuria (3–5). Sustained glomerular injury eventually progresses to end-stage kidney disease (ESKD), requiring dialysis or kidney transplantation (6).

Hematuria can manifest as microscopic (>5 red blood cells per high-power field [RBC/HPF]) or macroscopic (visible in the urine) (7). Microhematuria is the most common clinical manifestation of IgAN, reported in 70% to 100% of patients (8),and is associated with inflammatory and necrotizing kidney lesions. Macrohematuria is rarer in adults, more common in children, and may indicate a distinct disease phenotype (9). In the few adults who experience macrohematuria, it is usually concurrent with respiratory or gastrointestinal infections and may prompt earlier clinical intervention (7, 9–16).

To better characterize the magnitude and consistency of the association between hematuria and long-term kidney outcomes in IgAN, He et al. conducted a systematic review and meta-analysis of 13 observational studies comprising more than 5,000 patients with IgAN (17). They found that initial microhematuria (a single measurement) was associated with an 87% increased risk of ESKD and that persistent hematuria may represent an independent risk factor for poor kidney outcomes. While these findings offer valuable insights into the prognostic relevance of hematuria in IgAN, they are based on literature available through May 2020. Since then, a substantial body of new research has emerged, underscoring the need for an updated synthesis on the association between hematuria and long-term kidney outcomes in IgAN. This study therefore aims to expand and update the existing body of evidence on the association between hematuria and long-term kidney outcomes, evaluating the prognostic relevance of hematuria through a systematic literature review (SLR) and meta-analysis incorporating more recent studies.

## Materials and methods

### Systematic literature review

An SLR was conducted to identify studies evaluating the association between hematuria and kidney outcomes in patients with IgAN. The review was performed according to University of York Centre for Reviews and Dissemination (CRD) guidance and reported per Preferred Reporting Items for Systematic Reviews and Meta-Analyses (PRISMA) guidelines (18, 19).

### Data sources and search strategy

Searches were conducted in PubMed/MEDLINE and Embase databases (**Supplementary Table 1**). Searches were conducted for studies published from January 1, 2005, to June 20, 2025 (until March 20, 2025 for PubMed), corresponding to the period during which currently recommended treatments, including angiotensin receptor blockers and angiotensin-converting enzyme inhibitors have been on the market. Searches for conference abstracts were limited to those published between 2023 and 2025. The bibliographies of relevant SLR and included studies were hand-searched to identify additional citations of interest. The search strategy was developed using database-specific controlled vocabulary, as well as free-text terms, and was validated against a sample of published studies that would be expected to be identified for the review (**Supplementary Table 2**). The literature search was not updated before submission of the manuscript.

### Eligibility criteria

Studies were selected based on predetermined Population, Exposure, Outcomes, and Study Design (PEOS) criteria. Eligible publications comprised English-language studies that enrolled adults (≥18 years) with IgAN and reported ≥1 kidney outcome of interest, including changes in estimated glomerular filtration rate (eGFR), ESKD development, dialysis, kidney transplant, mortality, and clinical remission. Study types comprised clinical trials and *post-hoc* analyses thereof; observational/real-world evidence studies including prospective cohort and cross-sectional studies, retrospective analyses of real-world databases, disease registries, and case-control studies; conference abstracts; and meta-analyses. Non-peer-reviewed publications, commentaries, narrative reviews, and clinical guidelines were excluded.

### Study selection

**Figure 1:**
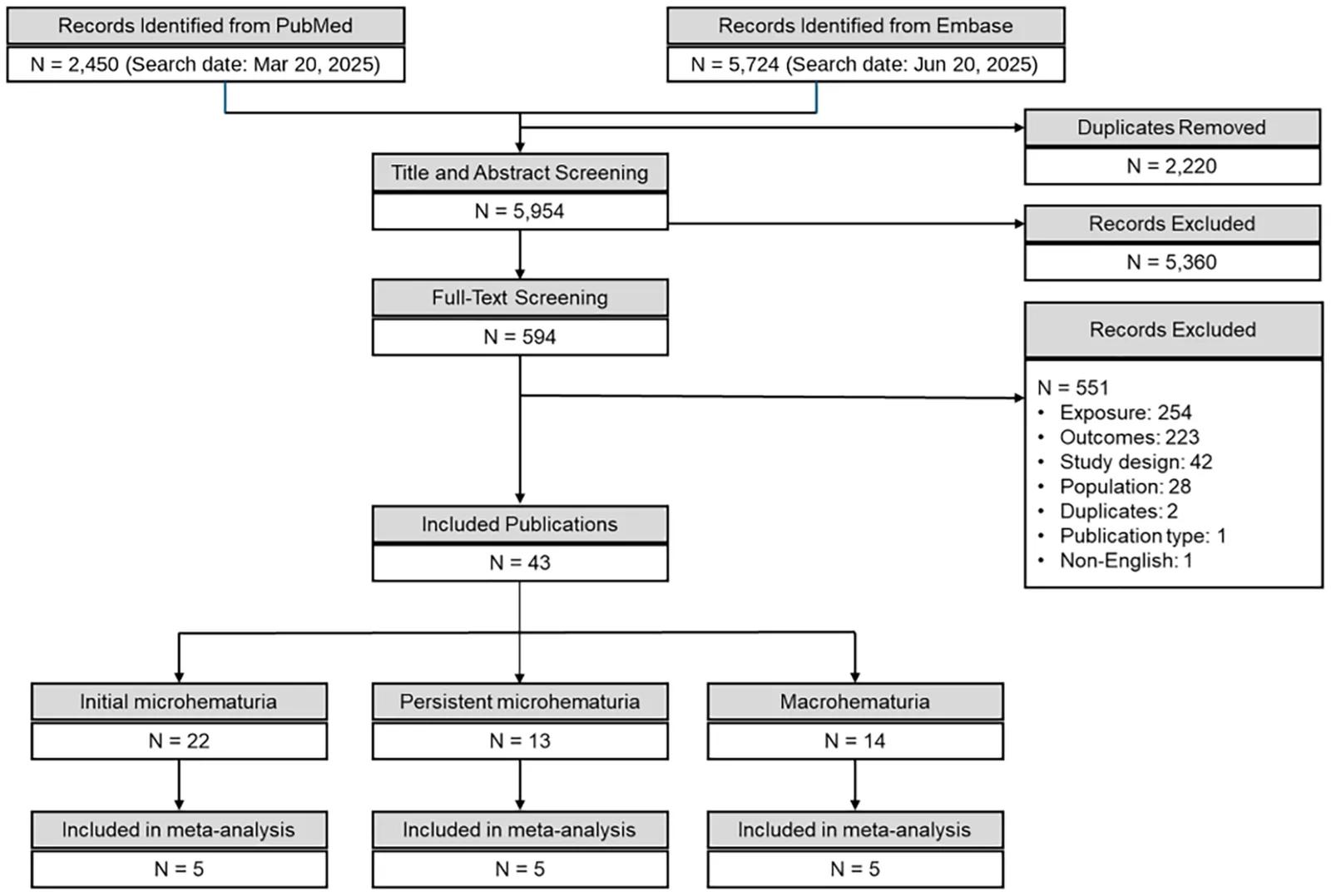
PRISMA flow diagram of literature identified via an SLR and its eligibility for meta-analysis. PRISMA, Preferred Reporting Items for Systematic Reviews and Meta-Analyses; SLR, systematic literature review.

Citations identified through the searches were exported to EndNote 20, deduplicated, and then uploaded to DistillerSR^®^ for screening. Study selection was performed in two phases: title/abstract screening and full-text review. Each citation was independently assessed by two reviewers and discrepancies were resolved through discussion, with unresolved conflicts adjudicated by a third, senior reviewer. Reasons for exclusion at the full-text stage were recorded. A PRISMA flow diagram was constructed to summarize the study identification and selection process (**Figure 1**) (18).

### Data extraction

Data were extracted into a standardized Excel^®^ grid by one reviewer and independently verified by a second reviewer, with discrepancies resolved by a third reviewer through consensus. Extracted data comprised study information (e.g., design, period, location, and sample size), patient demographics and baseline clinical characteristics (e.g., age, sex, race, and kidney function measures including eGFR and serum creatinine [SCr]), and definitions of hematuria (e.g., thresholds, timing, and assessment protocols).

Kidney failure outcomes extracted included those relating to ESKD (e.g., ESKD, dialysis, and transplant) and composite or individual endpoints reflecting progression to kidney failure (ESKD, initiation of chronic dialysis, kidney transplantation, and sustained eGFR <15 mL/min/1.73 m²). Kidney progression outcomes reflecting clinically meaningful decline in kidney function prior to failure were also extracted; they included doubling or other increase in SCr from baseline, sustained eGFR decline (e.g., ≥30%, ≥40%, or ≥50%), and annualized eGFR slope.

Data were extracted on estimates of the association between hematuria and kidney outcomes, including the type of analysis and reported measures of uncertainty (e.g., confidence intervals (CI) and standard errors). Coefficients were oriented so that, for binary variables, values >1 and, for continuous variables, values >0 indicate an association between hematuria and worse kidney outcomes. Where available, adjusted estimates were prioritized over unadjusted estimates.

### Quality assessment

The quality of included studies was evaluated using study design-specific appraisal tools, including the National Institute for Health and Care Excellence (NICE) checklist for the Newcastle-Ottawa Quality Assessment scale (NOS) for observational studies (**Supplementary Table 3**) (20). Each study could receive a total NOS score between 0–9 points based on the following domain scoring system: 1) up to 4 points for selection of the study population, 2) up to 2 points for comparability of participants, and 3) up to 3 points for the outcome. Additional tools were applied as appropriate to other included study designs.

### Evidence synthesis

#### Exposure classification

Studies identified through the SLR reported associations that were categorized into mutually exclusive hematuria groups independent of hematuria laboratory test used: (i) persistent microhematuria (≥2 time points); (ii) initial microhematuria (at single time point during the patient journey); or (iii) macrohematuria (measured as either a single time point or persistent). Analyses were conducted for microhematuria overall (combining persistent and initial) and separately for each category (persistent, initial, and macrohematuria). Institutional review board approval was not required for the present study, as this study was based solely on analysis of data from previously published studies and did not involve primary data collection or identifiable patient-level information.

#### Qualitative synthesis

Study estimates were classified as ‘increased risk’, ‘decreased risk’, or ‘not statistically significant’ based on a p-value <0.05 and corresponding CIs. The proportion of estimates in each category was summarized by hematuria definition.

#### Meta-analysis

Publications identified through the SLR were assessed for meta-analysis based on data availability, methodological comparability, and clinical relevance. Key reasons for exclusion included absence of relevant effect estimates, reporting of results on non-comparable scales (e.g., linear coefficients or odds ratios), inconsistent or non-comparable definitions of hematuria, and kidney outcomes not including ESKD. Full details on exclusion reasons are provided in **Supplementary Table 4**.

### Statistical analysis

The outcome of interest for the meta-analysis was relative hazards of ESKD-inclusive outcomes across patient cohorts defined by hematuria categories (all microhematuria, persistent microhematuria only, initial microhematuria only, and macrohematuria). ESKD-inclusive outcomes were defined as outcomes containing ESKD, dialysis, kidney transplantation, sustained eGFR <15 mL/min/1.73 m², or composite kidney failure outcomes including one or more of these components. Hazard ratios (HRs) and corresponding measures of variance (e.g., standard error or CIs) comparing kidney outcomes between hematuria-defined patient cohorts were pooled using random-effects meta-analyses with inverse-variance weighting, restricted maximum likelihood (REML) estimation, and Knapp-Hartung adjustment (21, 22). Statistical heterogeneity was assessed using the I² statistic and Cochran’s Q test, and results were presented using forest plots showing individual study estimates and pooled effect sizes.

## Results

### Systematic literature review

#### Overview of included studies

The SLR identified 43 eligible publications (**Figure 1**) reporting hematuria and kidney outcomes in adult IgAN. Studies were published between 2005 and 2025, with a median publication year of 2020. Thirty-three studies including 16,460 patients with IgAN reported on the effect of microhematuria (both single time-point and persistent), of which thirteen studies reported information on persistent microhematuria (6,134 patients, **Table 1**) (23–35) and 22 studies on initial microhematuria (11,094 patients, **Table 2**) (23, 25, 36–54); 14 studies including 8,477 patients reported on macrohematuria where all but one study measured macrohematuria at a single time point (**Table 3**) (13, 28, 34, 36, 43, 56–64). Six of these studies reported data on multiple hematuria categories (23, 25, 28, 34, 36, 43).

**Table 1:** Characteristics of studies identified via SLR evaluating persistent microhematuria and kidney outcomes in IgAN (n=13).

Study	N	Country	Outcomes	Definition of Hematuria	HR (95% CI) or other coefficient	Association with kidney outcomes
Bobart, 2021 (23)	72	USA	Rate of decline in eGFR	Median hematuria during follow-up >21 RBCs/HPF	Linear Coefficient, GEE Model -1.39 (-7.98, -2.52) ^1^	Worse outcomes
Huang, 2023 (24)	684	China	>30% eGFR, ESKD or death	Persistent hematuria: baseline and follow-up uRBC >24 RBC/μL High vs. low: based on baseline uRBC (cutoff 330 RBC/μL; tertiles)	3.93 (1.33, 11.6)	Worse outcomes
Koike, 2024 (25)	696	Japan	≥50% increase in SCr or dialysis (patients <20 at biopsy: dialysis or ≥25% eGFR decline)	Time-dependent remission of 5 RBC/HPF	3.45 (1.82, 6.67)	Worse outcomes
Le, 2012 (26)	1,155	China	≥50% eGFR or ESKD	TA-P Area Under the Curve (continuous variable)	2.1 (1.6, 2.7)	Worse outcomes
Sevillano, 2017 (27)	112	Spain	ESKD (eGFR <15 mL/min/1.73 m2, dialysis, or transplantation)	TA-hematuria of >5 RBC/HPF	2.84 (1.06, 7.3)	Worse outcomes
Weng, 2022 (28)	152	China	2× SCr level or ESKD (eGFR <15 mL/min/1.73 m2)	TA-hematuria area under the curve (continuous variable)	1.004 (1.001, 1.008)	Worse outcomes
Yu, 2020 (29)	1,333	China	≥50% eGFR decline or kidney failure	TA-Hematuria >5 RBC/HPF (manual)	2.27 (1.54, 3.33)	Worse outcomes
Ma, 2020 (30)	338	China	ESKD	Positive test for urine occult blood	3.08 (1.12, 8.48)	Worse outcomes
Li, 2022 (31)	452	China	ESKD or 100% increase in SCr	Remission: Occult blood ranging from (−) to (±) on three consecutive visits over 6 months	1.25 (0.15, 10.32)	NS
Yano, 2024 (32)	889	Japan	≥50% eGFR	High-stable vs low-stable patients based on longitudinal urine dipstick	2.69 (1.54, 4.7)	Worse outcomes
Yasuda, 2024 (33)	100	Japan	30% eGFR decline	Remission: < 5 RBC/HPF in three consecutive urine exams over six months of observation	16.67 (2.78, 100)	Worse outcomes
Ebbestad, 2022 (34)	77	Sweden	50% eGFR or ESKD (eGFR <15 mL/min/1.73 m2, dialysis, transplant)	> 10 RBC/HPF	NR	NS
Matsuzaki, 2021 (35)	74	Japan	eGFR per year	Three consecutive dipstick test results of (−) to (±) or a red blood cell count <5 in urinary sediment per high-power field during at least 6 months	Linear coefficient: -0.48 (-2.37, 1.41)	NS

CI, confidence interval; eGFR, estimated glomerular filtration rate; ESKD, end-stage kidney disease; GEE Model, Generalized Estimating Equations Model; HPF, high-power field; HR, hazard ratio; m2, square meter; IgAN, immunoglobulin A nephropathy; μL, microliter; mL, milliliter; min, minute; NR, not reported; RBC, red blood cells; SCr, serum creatinine; SLR, systematic literature review; TA, time-averaged; USA, United States of America.

Effect estimates were inverted where appropriate so that values >1 indicate an association with worse kidney outcomes.

**^1^**Estimate reported as published; apparent inconsistency between point estimate and 95% CI noted.

**Table 2:** Characteristics of studies identified via SLR evaluating initial microhematuria and kidney outcomes in IgAN (n=22).

Study	N	Country	Outcomes	Definition of Hematuria	HR (95% CI) or other coefficient	Association with kidney outcomes
Koike, 2024 (25)	696	Japan	≥50% increase in SCr or dialysis (patients <20 at biopsy: dialysis or ≥25% eGFR decline)	>20/HPF	0.66 (0.40, 1.09)	NS
Bobart, 2021 (23)	72	USA	ESKD	>3 RBCs/HPF	1.46 (0.18, 11.81)	NS
Goto, 2009 (36)	2,283	Japan	ESKD	1–29 RBC/HPF vs 0	2.34 (1.64, 3.33)	Worse outcomes
Manno, 2007 (37)	437	Italy	ESKD	Microhematuria at onset with absence of recurrent macrohematuria (yes vs no)	2.18 (1.30, 3.65)	Worse outcomes
Rivedal, 2025 (38)	400	Norway	ESKD or ≥50% eGFR decline	Urine dipstick	1.05 (0.38, 2.93)	NS
Zhang, 2024 (39)	1,512	China	CKD progression; baseline urinary RBC	Per 1 RBC/μL increase	1.001 (1.000, 1.001)	Worse outcomes
Nagai, 2022 (40)	133	Japan	2-year eGFR slope	Severe vs mild hematuria	Linear mixed-effects model: 1.44 ± 3.20 (severe) vs −0.52 ± 1.97 (mild)	Improved outcomes
Tan, 2022 (41)	126	China	50% eGFR decline, ESKD, or death	Per 1 RBC/HPF increase	1.003 (1.000, 1.006)	NS
Iwasaki, 2016 (42)	75	Japan	ESKD progression	Per 20 RBC/HPF increase	0.75 (0.55, 1.00)	NS
Tanaka, 2015 (43)	88	Japan	Proteinuria increase or treatment initiation	>20/HPF	1.06 (0.80, 1.36)	NS
Rafalska, 2014 (44)	52	Poland	Annual eGFR decline velocity	≥5 RBC/HPF	NR	Worse outcomes
Shen, 2007 (45)	82	China	eGFR <60 mL/min/1.73 m²	NR	OR: 2.87 (NR)	Worse outcomes
Shen, 2008 (47)	177	China	eGFR <60 mL/min/1.73 m²	>3 RBC/HPF	OR: 2.73 (NR)	Worse outcomes
Shen, 2010 (46)	135	China	Doubling of SCr from baseline	>3 RBC/HPF	OR: 2.97 (1.34, 5.13)	Worse outcomes
Neves, 2020 (48)	111	Brazil	ESKD or doubling of SCr from baseline	≥5 RBC/HPF	OR: 0.61 (0.26, 1.44)	NS
Bednarova, 2023 (49)	442	Czech Republic	Allograft failure	>10/μL at biopsy	2.49 (1.32, 4.69)	Worse outcomes
Liu, 2021 (50)	246	China	eGFR <60 at end of follow-up	Per RBC/HPF	OR: 1.002 (1.001, 1.003)	Worse outcomes
Liu, 2023 (51)	50	China	Annual rate of eGFR decline	Hematuria severity	Linear coefficient: 0.000 (-0.056, 0.056)	NS
Miyabe, 2021 (52)	871	Japan	ESKD	Per 25/HPF increase	0.98 (0.85, 1.12)	NS
Rui, 2022 (53)	101	China	ESKD or death	Per μL	1.00 (0.999, 1.00)	NS
Xing, 2024 (54)	736	China	SCr doubling, 40% eGFR decline, ESKD, or death	NR	OR: 0.999 (0.993, 1.004)	NS
Wakai, 2006 (55)	2,269	Japan	ESKD (initiation of dialysis)	0 RBCs/HPF, 1–29 RBCs/HPF, >30 RBCs/HPF	RR for 1–29 vs 0 RBC/HPF: 3.65 (2.02, 6.60); ≥30 vs 0 RBC/HPF: 1.37 (0.65, 2.89)	NS

CI, confidence interval; CKD, chronic kidney disease; eGFR, estimated glomerular filtration rate; ESKD, end-stage kidney disease; HPF, high-power field; HR, hazard ratio; IgAN, immunoglobulin A nephropathy; μL, microliter; mL, milliliter; min, minute; NR, not reported; NS, not statistically significant; OR, odds ratio; RBC, red blood cells; RR, relative risk; SCr, serum creatinine; SLR, systematic literature review; USA, United States of America.

Effect estimates were inverted where appropriate so that values >1 indicate an association with worse kidney outcomes.

**^1^**Estimate reported as published; apparent inconsistency between point estimate and 95% CI noted.

**Table 3:** Characteristics of studies identified via SLR evaluating macrohematuria and kidney outcomes in IgAN (n=14).

Study	N	Country	Outcomes	Timepoint of Macrohematuria assessment	HR (95% CI) or other coefficient	Association with kidney outcomes
Kovács, 2014 (56)	264	Hungary	eGFR <30 ml/min/1.73 or ESKD	Renal biopsy	eGFR: 0.63 (0.35, 1.16) ESKD: 0.48 (0.22, 1.04)	NS
Le, 2014 (13)	1,155	China	ESKD or ≥50% eGFR decline	No MH, isolated MH (i.e., one episode), recurrent MH (2 or more episodes): Follow-up or history	Recurrent MH: Survival from biopsy: 1.10 (0.72, 1.68) Survival from disease onset: 0.65 (0.42, 0.99)	Improved outcomes
Lee, 2012 (57)	1,364	Korea	ESKD	Baseline^a^	0.61 (0.41, 0.91)	Improved outcomes
Pan, 2018 (58)	825	China	ESKD or ≥50% eGFR decline	History of MH	0.02 (0.00, 0.54)	Improved outcomes
Goto, 2009 (36)	2,283	Japan	ESKD	Baseline	0.45 (0.26, 0.77)	Improved outcomes
Zhang, 2016 (59)	1,512	China	AKI	Baseline^a^	0.41 (0.24, 0.70)	Improved outcomes
Espinosa, 2009 (60)	59	Spain	ESKD	Renal biopsy	Difference in % with ESKD vs % without ESKD: 52.3%	Improved outcomes
Chacko, 2005 (61)	347	India	≥20% eGFR decline	Renal biopsy	OR: 0.23 (0.47, 1.15)^b^	NS
Weng, 2022 (28)	152	China	Doubling of baseline SCr level or ESKD (eGFR <15 ml/min/1.73)	Renal biopsy	0.59 (0.10, 3.53)	NS
Gutiérrez, 2007 (62)	32	Spain	≥25% from baseline SCr	History of MH	OR: 0.98 (0.96, 0.99)	Improved outcomes
Shu, 2017 (63)	150	China	ESKD (eGFR <15 ml/min/1.73 or initiation of dialysis or transplantation)	Renal biopsy	0.05 (0.00, 9.02)	NS
Mahanta, 2019 (64)	169	India	AKI	Baseline^a^	OR: 4.21 (1.11, 15.9)	Worse outcomes
Ebbestad, 2022 (34)	77	Sweden	ESKD or ≥50% eGFR decline from baseline or initiation of dialysis or transplantation	History of MH	NR	NS
Tanaka, 2015 (43)	88	Japan	Increasing proteinuria to 1 g/day or starting treatment with corticosteroids, immunosuppressive agents, or undergoing tonsillectomy	Baseline^a^	0.85 (0.22, 2.96)	NS

AKI, acute kidney injury; CI, confidence interval; eGFR, estimated glomerular filtration rate; ESKD, end-stage kidney disease; HPF, high-power field; HR, hazard ratio; m2, square meter; μL, microliter; mL, milliliter; min, minute; NR, not reported; OR, odds ratio; RBC, red blood cells; SCr, serum creatinine; MH: macrohematuria.

Effect estimates were inverted where appropriate so that values >1 indicate an association with worse kidney outcomes. ^a^The timing of the macrohematuria assessments were not clearly reported in these studies. ^b^Estimates are shown as reported.

Across the 43 studies, sample sizes ranged from 32 to 2,283, with a median of 246. Median sample sizes were 156 for studies of initial microhematuria, 338 for persistent microhematuria, and 217 for macrohematuria. Most studies were conducted in China (n=19) and Japan (n=10), with additional contributions from Spain (n=3), India (n=2), and single studies from the United States, Brazil, Italy, Sweden, Poland, the Czech Republic, Hungary, Norway, and South Korea.

Microhematuria definitions were heterogeneous across studies but generally fell into three categories. First, threshold-based measures were most common, typically defined as 3–10 RBC/HPF, although some studies used higher cut-offs or equivalent RBC/μL values. Second, longitudinal definitions captured hematuria over time, including time-averaged measures (e.g., area under the curve) or remission-based definitions, such as sustained thresholds or persistently negative dipstick results. Third, alternative approaches were used in some studies, including continuous measures (e.g., per-unit increase in RBCs), dipstick-based definitions, or categorization by severity levels.

The most commonly reported long-term kidney outcomes were ESKD development (21 studies reporting ESKD alone and 5 reporting composite outcomes including ESKD) and changes in eGFR or serum creatinine (23 studies). Six studies reported other outcomes such as CKD progression.

Overall study quality, assessed using the NOS, was generally good across studies (median score: 7 [IQR: 6–8] out of 9), although variability was observed across individual domains (**Supplementary Table 3**). Methodological limitations were observed in the domains for comparability and outcome. Studies with limitations on comparability did not adequately adjust for established prognostic markers such as blood pressure, histologic MEST-C features, or treatment exposure. Consequently, the impact of hematuria may be a biased estimate due to residual confounding in these studies. Studies that had lower scores in the outcome domain had variable follow-up duration that could have been insufficient to capture long-term kidney endpoints. These factors could have introduced attrition and outcome ascertainment bias. (**Supplementary Table 3**).

Most studies utilized multivariable models: 11 of 13 (85%) studies analyzing persistent microhematuria, 12 of 22 (55%) studies analyzing any microhematuria, and 8 of 14 (57%) studies analyzing macrohematuria. Adjustment sets varied across studies; among multivariable models, covariates most commonly included demographic factors (age, sex/gender), baseline kidney function (UPCR, UPER, eGFR, serum creatinine), proteinuria, blood pressure or hypertension, histologic features (MEST-C score – total or individual components, time since biopsy), and treatment exposure (RASi/ACEi/ARB use, steroid/prednisone/immunosuppressant use, SGLT2i use), whereas several studies (persistent microhematuria: n=2; initial microhematuria: n=10; macrohematuria: n=6) reported univariable estimates only or did not report adjustment variables (**Supplementary Table 5**).

#### Association of hematuria with kidney outcomes

**Figure 2:**
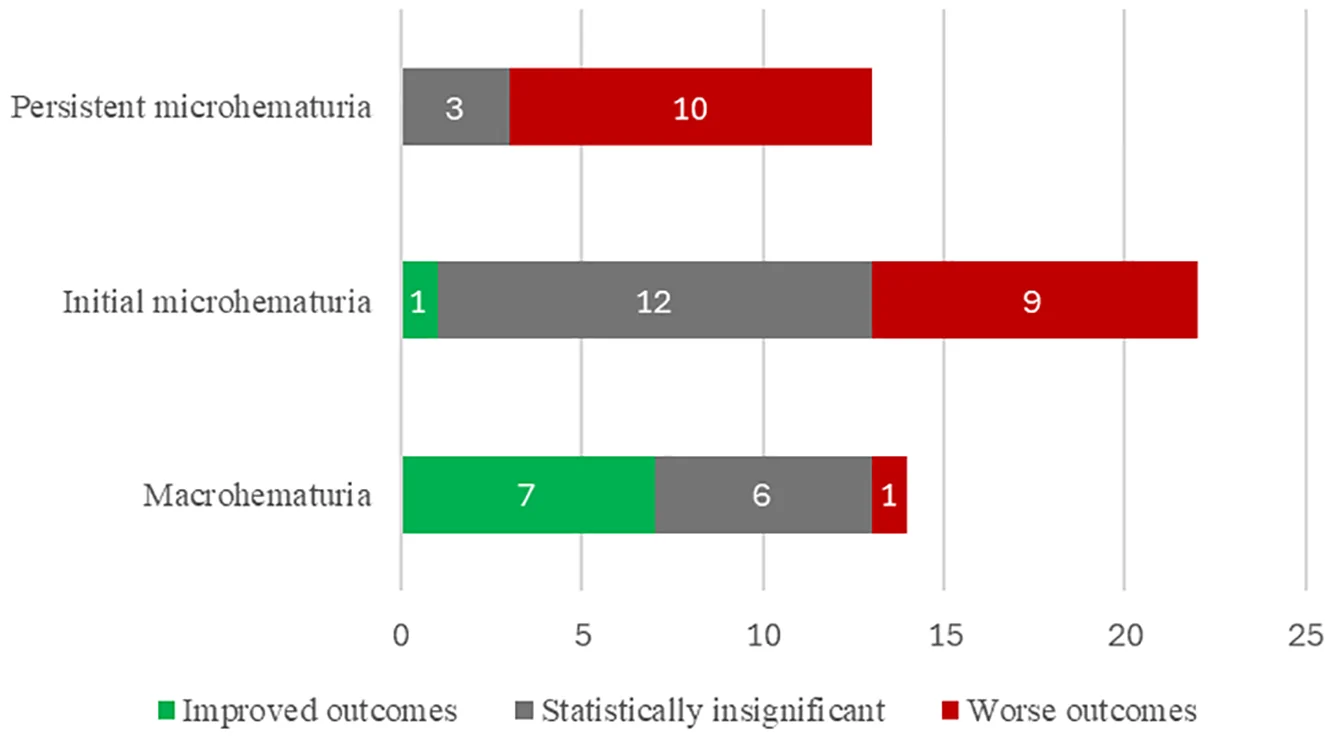
Number of studies evaluating the association between hematuria and kidney outcomes. Studies reported associations of hematuria with either improved, worse, or not statistically different kidney outcomes based on p<0.05 or corresponding confidence intervals.

Across 33 unique articles evaluating microhematuria, ten of 13 studies in the persistent microhematuria group (representing 5,531 of the total 6,134 patients) and nine of 22 studies (representing 5,366 of the total of 11,094 patients) in the initial microhematuria group found a significant association between hematuria and increased risk of kidney outcomes (**Figure 2**); one study (133 patients) found an association of initial microhematuria with improved outcomes; the remaining studies (three studies including 603 patients on persistent microhematuria and 12 studies including 5,595 patients on initial microhematuria) found no association (**Tables 1**, **2**).

Among the 14 articles reporting results on macrohematuria (all but one study measuring macrohematuria at a single time point), seven studies (representing 7,230 of a total of 8,477 patients) reported an association of hematuria with improved outcomes, six studies (1,078 patients) found no association, and one study (169 patients) found an increased risk of kidney outcomes (**Table 3**).

#### Kidney survival

Lower time averaged (TA)-microhematuria was associated with favorable kidney survival, independent of proteinuria status. In a retrospective cohort study conducted in China (N = 152; mean follow-up, 58.08 months), kidney survival rates were significantly higher among patients with less severe TA-microhematuria than in those with more severe TA-microhematuria (log-rank p=0.049) (28). In analyses stratified by both hematuria and proteinuria status, patients with hematuria remission and proteinuria remission had the most favorable kidney outcomes, whereas those with persistent hematuria had poorer outcomes (overall log-rank p=0.001).

#### Kidney functional decline

A slower decline in kidney function was reported for patients with microhematuria remission. In a cohort from Spain (N = 112) with a mean follow-up of 14 years, microhematuria resolved in 46% of patients with a median time to remission of 5.98 years. Among these patients, kidney function decline was significantly slower than before remission (eGFR slope, −0.18 ± 2.56 vs −6.45 ± 14.66 mL/min/1.73 m²/year; p=0.001) (27).

Microhematuria remission was also associated with a lower risk of kidney disease progression, defined as a ≥50% eGFR decline or kidney failure, irrespective of the type of assessment (automated or manual) of microhematuria remission. In one study (29), HR was reported comparing patients with microhematuria remission versus those without remission. Using an automated method with a cutoff of 28 RBC/μL, the HR for 50% eGFR decline or kidney failure was 0.45 (95% CI, 0.32–0.64) in the unadjusted analysis and 0.41 (95% CI, 0.28–0.61) after adjustment for age, sex, log-transformed proteinuria, mean arterial pressure, and steroids or other immunosuppressive agents (all p<0.001). Results were similar with manual assessment with a cutoff of 5 RBC/HPF, with corresponding hazard ratios of 0.49 (95% CI, 0.35–0.68) and 0.44 (95% CI, 0.30–0.65); all p<0.001 (29).

### Meta-analysis

A total of 15 study entries reporting HRs for ESKD-inclusive outcomes were included in the meta-analysis, corresponding to 13 unique publications (13, 23, 25, 27, 29–31, 36–38, 56–58), as two studies (25, 36) contributed data for more than one hematuria category.

**Figure 3:**
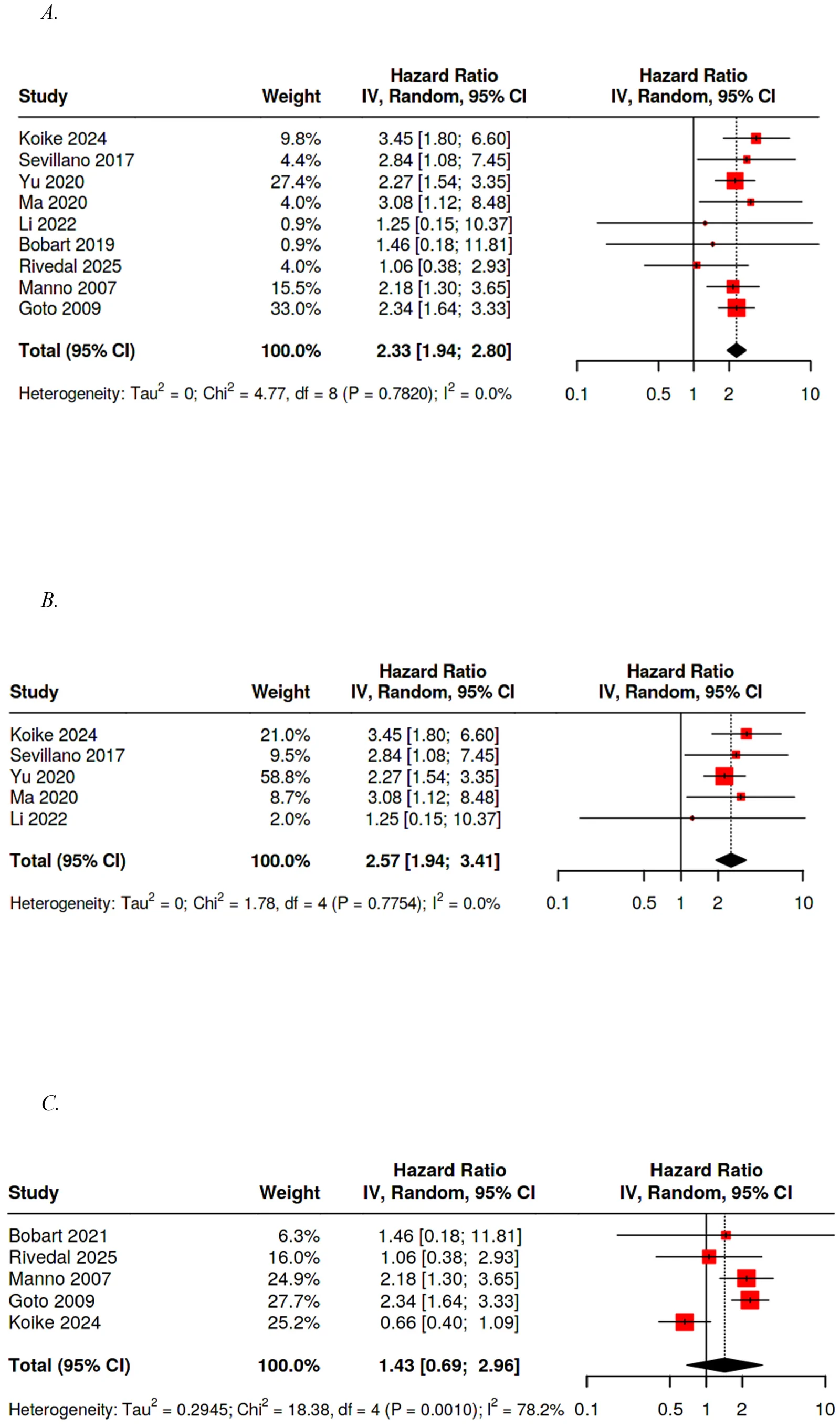
Forest plot of pooled hazard ratios for microhematuria. **(A)** Overall microhematuria. Chi², Cochran’s Q test statistic; CI, confidence interval; df, degrees of freedom; HR, hazard ratio; IV, inverse variance; I², heterogeneity statistic; Tau², between-study variance. HR and 95% CIs are derived from log-transformed estimates and standard errors calculated from the reported values; small differences from published values may occur due to rounding. **(B)** Persistent microhematuria. Chi², Cochran’s Q test; CI, confidence interval; df, degrees of freedom; HR, hazard ratio; IV, inverse variance; I², heterogeneity statistic; Tau², between-study variance. HR and 95% CIs are derived from log-transformed estimates and standard errors calculated from the reported values; small differences from published values may occur due to rounding. **(C)** Initial microhematuria. Chi², Cochran’s Q test statistic; CI, confidence interval; df, degrees of freedom; HR, hazard ratio; IV, inverse variance; I², heterogeneity statistic; Tau², between-study variance. HR and 95% CIs are derived from log-transformed estimates and standard errors calculated from the reported values; small differences from published values may occur due to rounding.

Across nine studies, microhematuria (either persistent or initial) was associated with a significant (p<0.05) increase in risk of ESKD-inclusive outcomes with no between-study heterogeneity (HR, 2.33; 95% CI, 1.94–2.80; I²=0%) (**Figure 3**). Among studies evaluating persistent microhematuria (n=5) (25, 27, 29–31), the association with ESKD-inclusive outcomes remained consistent with that observed in the overall microhematuria analysis (HR, 2.57; 95% CI, 1.94–3.41). Study-level effect estimates were uniformly >1.0, and between-study heterogeneity was not observed (I²=0%), supporting the robustness of the pooled finding (**Figure 3**). Initial microhematuria (n=5) (23, 25, 36–38) was associated with a non-significant increased risk (HR 1.43, 95% CI 0.69–2.96), although the substantial between-study heterogeneity (I²=78%) and variability in study-level effect estimates, limits confidence in this finding (**Figure 3**).

**Figure 4:**
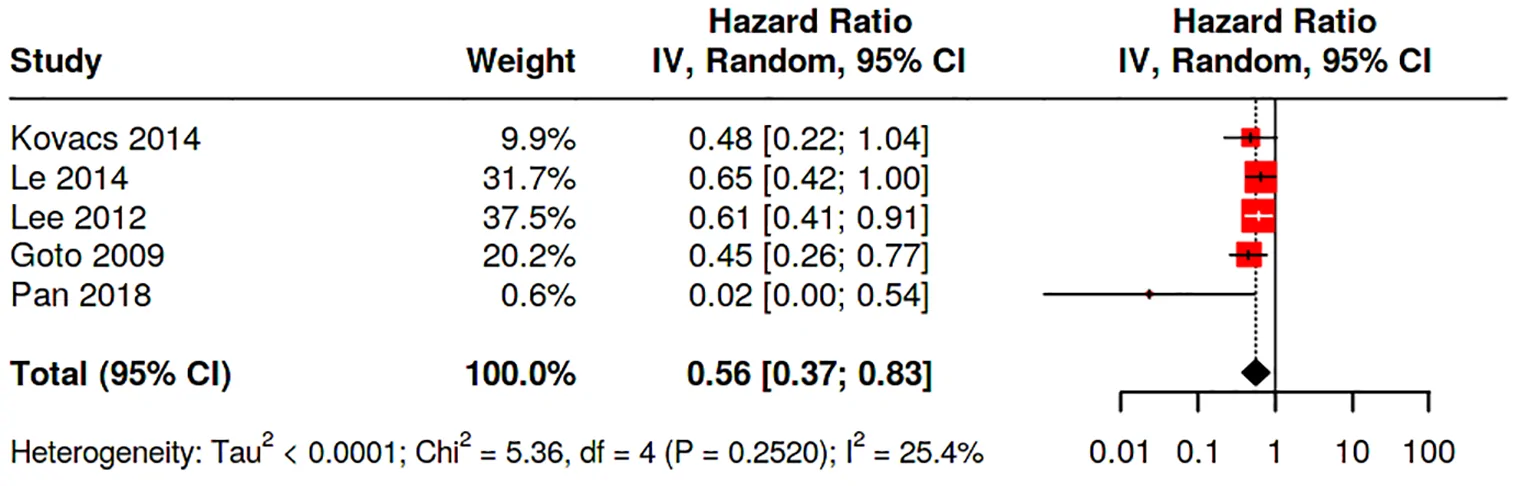
Forest plot of pooled hazard ratios for macrohematuria. Chi², Cochran’s Q test; CI, confidence interval; df, degrees of freedom; HR, hazard ratio; IV, inverse variance; I², heterogeneity statistic; Tau², between-study variance. HR and 95% CIs are derived from log-transformed estimates and standard errors calculated from the reported values; small differences from published values may occur due to rounding.

In contrast, macrohematuria (n=5) (13, 36, 56–58) was associated with reduced risk (HR 0.56, 95% CI 0.37–0.83), with all study-level effect estimates <1.0 and low heterogeneity (I^2^ = 25%) (**Figure 4**).

## Discussion

This systematic review and meta-analysis demonstrated consistent and clinically meaningful associations between presence of microhematuria and kidney failure outcomes in adults with IgAN. In the meta-analyses, microhematuria (pooled persistent and initial) was associated with a more than two-fold increased risk of ESKD-inclusive outcomes (HR, 2.33; 95% CI, 1.94–2.80). This association was driven by persistent microhematuria, which showed a similarly elevated risk (HR, 2.57; 95% CI, 1.94–3.41), with consistent study-by-study estimates and no observed heterogeneity. These findings are supported by the qualitative synthesis, in which the majority of studies evaluating persistent microhematuria (10/13 studies; 5,531/6,134 patients) reported a significant association with increased risk of kidney outcomes. A similar directional association was observed for initial microhematuria, suggesting that greater hematuria burden may increase the risk of worse kidney outcomes when assessed at a single time point. However, in contrast to persistent exposure, results were heterogeneous across studies and did not reach statistical significance (HR, 1.43; 95% CI, 0.69–2.96). Consistent with this, fewer studies in the qualitative synthesis reported significant associations for initial microhematuria (9/22; 5,366/11,094 patients), with most of the 13 remaining studies finding no association and one reporting a reduced risk (n=1). The greater heterogeneity likely reflects differences in baseline/initial hematuria definitions, measurement methods, and timing of assessment, as well as the possibility that a single measurement may capture urinary abnormalities less reliably than repeated assessments over time.

In this context, persistent microhematuria likely represents a prognostically informative marker of ongoing glomerular injury in IgAN, more closely reflecting sustained disease activity than initial microhematuria. This interpretation is consistent with the broader qualitative findings of our systematic review, in which individual studies evaluating TA-H, microhematuria remission, or persistent hematuria patterns consistently reported worse kidney outcomes with sustained hematuria burden over time.

By contrast, our meta-analysis found macrohematuria (predominantly measured at a single timepoint) to be associated with improved ESKD-related outcomes (HR 0.56, 95% CI 0.37–0.83), although this finding should be interpreted cautiously. This estimated association was consistent with the qualitative synthesis, in which half of the included studies (7/14; 7,230/8,477 patients) reported an association of hematuria with improved outcomes, while most remaining studies found no association and only one reported an increased risk. Rather than suggesting macrohematuria measured at a single point in time is associated with longer kidney survival, the association may reflect differences in patient phenotype, timing of hematuria measurement, and downstream clinical management. For example, Zhang et al. (2016) found macroscopic hematuria to be associated with lower odds of acute kidney injury (AKI) in univariable analysis and suggested that macrohematuria may prompt earlier clinical detection and treatment (59). Le et al. (2014) similarly reported more favorable long-term outcomes among patients with recurrent macroscopic hematuria, but these patients appeared to represent a distinct IgAN phenotype, characterized by younger age, less hypertension, better kidney function, and less chronic histologic injury (13). Pan et al. (2018) found macrohematuria to be independently associated with lower risk of poor renal outcomes among IgAN patients with segmental glomerular necrosis, a group that more often received immunosuppressive therapy, raising the possibility that hematuria may identify a more active, treatment-responsive inflammatory phenotype rather than irreversible chronic kidney damage (58). Thus, the observed favorable association may reflect earlier presentation, lower baseline chronicity, and treatment-responsive disease, rather than a direct beneficial effect of macrohematuria.

Our findings are consistent with prior evidence supporting the prognostic relevance of microhematuria in IgAN (7, 8, 24, 28, 29, 32, 65, 66). However, meta-analyses evaluating the impact of hematuria on kidney outcomes in IgAN remain scarce. The most recent was published in 2021 by He et al., who reported that initial microhematuria was associated with an 87% increased risk of ESKD (risk ratio, 1.87; 95% CI, 1.40–2.50) and identified persistent hematuria as an adverse prognostic factor for kidney outcomes. In our analysis, microhematuria was similarly associated with a significantly increased hazard of ESKD-inclusive outcomes overall (HR, 2.33; 95% CI, 1.94–2.80), with the strongest association observed for persistent microhematuria (HR, 2.57; 95% CI, 1.94–3.41). The present SLR and meta-analysis provide a substantially expanded evidence base, encompassing 43 publications (including 19 published after 2020) and 6,134 to 11,094 patients across analyses, compared with the 13 publications (5,660 patients) from 2020 or earlier included in He, et al.

This study has several strengths. The systematic review was conducted using rigorous and reproducible methodology, in accordance with CRD and PRISMA guidelines (18, 19). Predefined PEOS-based eligibility criteria ensured consistent application of study inclusion and exclusion requirements during both title/abstract and full-text screening. The search strategy was validated against known relevant studies and supplemented by hand-searching reference lists to reduce the risk of missing key publications. In the meta-analysis, studies were grouped by hematuria definition to ensure comparability of exposures, and random-effects models with Knapp-Hartung adjustment were used to provide reliable pooled estimates while accounting for between-study variability. In addition, concordance between systematic review and the meta-analysis results reinforces the association between persistent microhematuria and adverse kidney outcomes in IgAN.

Limitations include the observational design of the included studies, precluding causal interpretation of the reported associations. However, overall study quality was generally good, with a median NOS score of 7. Although adjusted estimates were prioritized, residual confounding cannot be excluded. In addition, differences across studies in covariate adjustment and treatment exposure may have affected the estimated associations, as the specific variables included in adjusted models were not consistent. Definitions of hematuria varied in thresholds and measurement methods and, despite restricting meta-analyses to ESKD-inclusive outcomes, residual differences likely contributed to between-study heterogeneity, particularly for initial microhematuria (I²=78%). The small number of study entries (n=5) in each hematuria category restricted sensitivity analyses and reduced statistical power. In addition, although searches of relevant conference proceedings through June 2025 were included to help capture recent and emerging evidence, the literature search was not updated after the final search dates reported above; therefore, very recently published studies may not be captured in this review. However, the review is a comprehensive synthesis of the evidence available through June 2025 Finally, it is important to acknowledge that persistent microhematuria is inherently longitudinal and many studies assessed it as a time-averaged measure over follow-up that captures the impact of cumulative exposure. Future studies conducted using more robust time-varying Cox models may provide more precise estimates of the prognostic relevance of persistent microhematuria.

Notwithstanding the above limitations, our findings have important implications for risk stratification and longitudinal monitoring in IgAN. The present systematic review and meta-analyses addresses an important evidence gap by synthesizing the association between hematuria and long-term kidney outcomes in adult IgAN. Findings from this synthesis support the potential relevance of hematuria, particularly persistent microhematuria, and its incremental evidence in risk prediction beyond proteinuria, eGFR, and histologic markers in IgAN. Current guideline-based risk assessment and established IgAN risk prediction tools account for established clinical and histologic predictors such as proteinuria, eGFR, blood pressure, and MEST-C lesions. These tools do not yet incorporate hematuria as a validated prognostic marker even though hematuria is a defining clinical feature in IgAN (67).The prognostic signal was strongest and most consistent for persistent microhematuria. Unlike a single hematuria measurement, persistence over time may better capture sustained glomerular inflammation or residual disease activity, which could explain its stronger association with ESKD-inclusive outcomes. These findings suggest that longitudinal assessments of hematuria could be informative for IgAN risk stratification, particularly as a complement to established predictors such as proteinuria, eGFR, blood pressure, and histologic lesions.

## Conclusions

Persistent microhematuria was consistently associated with an increased risk of ESKD in adult IgAN, with more than a 2.5-fold higher hazard compared with no persistent microhematuria. Our findings support the prognostic relevance of sustained hematuria burden and suggest that reductions in hematuria over time are associated with more favorable kidney outcomes. More broadly, they reinforce the value of hematuria as a clinically meaningful marker for risk stratification and longitudinal monitoring in IgAN.

## Data Availability

The original contributions presented in the study are included in the article/**Supplementary Material**. Further inquiries can be directed to the corresponding author.

## Supplementary material

Supplementary Table 1 PEOS Criteria. CKD, chronic kidney disease; eGFR, estimated glomerular filtration rate; ESKD, end-stage kidney disease; ESRD, end-stage renal disease; IgAN, immunoglobulin A nephropathy; NR, not reported; RBC, red blood cells; SLR, systematic literature review. ^a^Studies with mixed populations and stratified data were included. ^b^Systematic literature reviews were included for search strategy validation and bibliography check purposes only and not for data extraction and summary.

Supplementary Table 2 Electronic Database Search Strategies. A. PubMed search strategy. B. Embase (via ) search strategy

Supplementary Table 3 Study quality assessment using the Newcastle–Ottawa Scale (NOS) across included studies and domains (selection, comparability, and outcome) NOS, Newcastle-Ottawa Scale. Each study was assessed across three domains: selection (maximum 4 stars), comparability (maximum 2 stars), and outcome (maximum 3 stars), for a total possible score of 9 stars. ★ indicates one star awarded; ★★ indicates two stars awarded (comparability domain only). A dash (-) indicates no star awarded. Higher scores indicate higher study quality.

Supplementary Table 4 Exclusion reasons for studies identified in the SLR and not included in the meta-analysis

Supplementary Table 5 Adjustment variables reported by included studies evaluating hematuria and kidney outcomes in IgAN ACEi, angiotensin-converting enzyme inhibitor; ARB, angiotensin receptor blocker; BMI, body mass index; BP, blood pressure; BUN, blood urea nitrogen; CKD, chronic kidney disease; eGFR, estimated glomerular filtration rate; GFR, glomerular filtration rate; HDL, high-density lipoprotein; IFTA, interstitial fibrosis and tubular atrophy; IgAN, immunoglobulin A nephropathy; LDL, low-density lipoprotein; MEST-C, mesangial hypercellularity, endocapillary hypercellularity, segmental glomerulosclerosis, tubular atrophy/interstitial fibrosis, and crescents; N/A, not applicable; NR, not reported; RAAS/RAS, renin-angiotensin-aldosterone system/renin-angiotensin system; RBC, red blood cell; RF-RG, risk factor and renal grade; SGLT2, sodium-glucose cotransporter-2; UPER, urinary protein excretion rate.
