# Supplementary material for: Impact of hematuria on long term kidney outcomes in adults with immunoglobulin A nephropathy: a systematic literature review and meta-analysis
Source: Front Nephrol. 2026 Aug 12;6:1899597. doi: 10.3389/fneph.2026.1899597 (PMC13506698; doi:10.3389/fneph.2026.1899597)
Supplement: Supplementary file 1 [file Supplementaryfile1.docx]

## SUPPLEMENTARY MATERIAL

### Table S1. PEOS Criteria

| Criteria | Inclusion | Exclusion |
| --- | --- | --- |
| Population | - Adult (≥18 years) patients diagnosed with IgAN regardless of comorbidities^a^ | - Non-IgAN populations - Pediatric patients (<18 years) with IgAN |
| Exposure | - Hematuria | - Exposure other than those described in the inclusion criteria |
| Outcomes | - Evidence showing the importance of hematuria with respect to   - eGFR   - ESKD/ESRD development   - Dialysis   - Kidney transplant   - Mortality   - Clinical remission   - Proteinuria | - Outcomes other than those described in the inclusion criteria |
| Study Design/ | - Clinical trials (including post-hoc analyses) - Observational/real-world evidence studies (prospective cohort and cross-sectional studies, retrospective analyses of real-world databases, disease registries, case-control studies) - Conference abstracts - Meta-analyses - For evidence on the threshold for a change in proteinuria measured by any methods that is considered clinically meaningful only, also include: expert opinions, clinical guidelines, narrative reviews | - Commentaries, letters, notes, editorials, expert opinions, erratums, government reports, reports from specialist societies - Case studies, case reports, case series - Clinical trial design/protocol - Pre-clinical and animal studies - Narrative reviews - Clinical guidelines - SLRs^b^ |
| Publication type | - Peer-reviewed publications - Conference abstracts | - Non-peer-reviewed publications |
| Limits | - Language: English - Full-text articles published in the last 20 years: January 1, 2005 to present - Abstracts published in the last 3 years: January 1, 2023 to present | - Language: Non-English - Full-text articles published in 2004 or before - Abstracts published in 2022 or before |

**Abbreviations**: CKD, chronic kidney disease; eGFR, estimated glomerular filtration rate; ESKD, end-stage kidney disease; ESRD, end-stage renal disease; IgAN, immunoglobulin A nephropathy; NR, not reported; RBC, red blood cells; SLR, systematic literature review.

^a^Studies with mixed populations and stratified data were included.

^b^Systematic literature reviews were included for search strategy validation and bibliography check purposes only and not for data extraction and summary.

### Table S2. Electronic Database Search Strategies

A. PubMed search strategy

| **Domain** | **No.** | **Search terms** |
| --- | --- | --- |
| **IgAN** | 1 | "glomerulonephritis, iga"[MeSH] |
|  | 2 | "glomerulonephritis"[MeSH:noexp] AND "immunoglobulin a"[MeSH] |
|  | 3 | "IgA" AND "nephropath*" |
|  | 4 | "iga glomerulonephritis"[tiab: ~3] OR "igan" |
|  | 5 | ("immunoglobulin a" OR "immunoglobulin IgA"[tiab: ~3]) AND ("nephropath*" OR "glomerulonephritis") |
|  | 6 | "Bergers disease" OR "Berger's disease" OR "Berger disease" |
|  | 7 | #1 OR #2 OR #3 OR #4 OR #5 OR #6 |
| **Hematuria** | 8 | "hematuria" OR "haematuria" |
| **IgAN and exposure and outcomes** | 9 | #7 AND #8 |
| **Animal studies** | 10 | ("Animals"[Mesh] NOT "Humans"[Mesh]) OR "animal"[ti] OR "mouse"[ti] OR "mice"[ti] OR "swine"[ti] OR "porcine"[ti] OR "murine"[ti] OR "sheep"[ti] OR "lambs"[ti] OR "pigs"[ti] OR "piglets"[ti] OR "rabbit"[ti] OR "rabbits"[ti] OR "cat"[ti] OR "cats"[ti] OR "dog"[ti] OR "dogs"[ti] OR "cattle"[ti] OR "bovine"[ti] OR "monkey"[ti] OR "monkeys"[ti] OR "trout"[ti] OR "marmoset"[ti] OR "in vitro"[ti] OR "in vivo"[ti] |
| **Irrelevant study types** | 11 | "Case Reports"[pt] |
| **Exclusions** | 12 | #10 OR #11 |
| **IgAN and exposure and outcomes after exclusions** | 13 | #9 NOT #12 |
| **English language** | 14 | #13 AND English[language] |
| **Published between 2005 and 2025** | 15 | #14 AND ("2005/01/01"[dp]:"2025/03/20"[dp]) |

B. Embase (via Embase.com) search strategy

| **Domain** | **No.** | **Search terms** |
| --- | --- | --- |
| **Population** | 1 | 'immunoglobulin a nephropathy'/exp |
|  | 2 | 'glomerulonephritis'/de AND 'immunoglobulin a'/exp |
|  | 3 | 'iga' AND 'nephropath*' |
|  | 4 | ('iga' NEAR/3 'glomerulonephritis') OR 'igan' |
|  | 5 | ('immunoglobulin a' OR ('immunoglobulin' NEAR/3 'iga')) AND ('nephropath*' OR 'glomerulonephritis') |
|  | 6 | 'berger$ disease' |
|  | 7 | #1 OR #2 OR #3 OR #4 OR #5 OR #6 |
| **Hematuria** | 8 | 'he$maturia' |
| **IgAN and exposure and outcomes** | 9 | #7 AND #8 |
| **Animal studies** | 10 | 'animal'/exp NOT 'human'/exp OR 'animal':ti OR 'mouse':ti OR 'mice':ti OR 'swine':ti OR 'porcine':ti OR 'murine':ti OR 'sheep':ti OR 'lambs':ti OR 'pigs':ti OR 'piglets':ti OR 'rabbit':ti OR 'rabbits':ti OR 'cat':ti OR 'cats':ti OR 'dog':ti OR 'dogs':ti OR 'cattle':ti OR 'bovine':ti OR 'monkey':ti OR 'monkeys':ti OR 'trout':ti OR 'marmoset':ti OR 'in vitro':ti OR 'in vivo':ti |
| **Case study/reports** | 11 | 'case report':ti OR 'case study':ti |
| **Exclusions** | 12 | #10 OR #11 |
| **IgAN and exposure and outcomes after exclusions** | 13 | #9 NOT #12 |
| **Conference abstracts** | 14 | #13 AND ('conference abstract':it OR 'conference paper':it OR 'conference review':it) |
| **Full-text articles from 2005 to 2025** | 15 | (#13 NOT #14) AND [2005-2025]/py |
| **Conference abstracts from 2023 to 2025** | 16 | #14 AND [2023-2025]/py |
| **Full-text articles and conference abstracts** | 17 | #15 OR #16 |
| **English language** | 18 | #17 AND [english]/lim |

### Table S3. Study quality assessment using the Newcastle–Ottawa Scale (NOS) across included studies and domains (selection, comparability, and outcome)

| **Study (Author, Year)** | **Selection (max 4 ★)** | | | | **Comparability (max 2 ★)** | **Outcome (max 3 ★)** | | | **Total NOS Score (/9)** |
| --- | --- | --- | --- | --- | --- | --- | --- | --- | --- |
|  | **Representativeness of exposed cohort** | **Selection of non-exposed cohort** | **Ascertainment of exposure** | **Outcome not present at start** |  | **Assessment of outcome** | **Length of follow-up** | **Adequacy of follow-up** |  |
| Bednarova, 2023 | ★ | ★ | ★ | ★ | ★ | ★ | ★ | - | **7** |
| Bobart, 2021 | ★ | ★ | ★ | - | ★ | ★ | - | - | **5** |
| Chacko, 2005 | ★ | ★ | ★ | ★ | - | ★ | ★ | - | **6** |
| Ebbestad, 2022 | ★ | ★ | ★ | ★ | - | ★ | ★ | ★ | **7** |
| Espinosa, 2009 | ★ | ★ | ★ | - | ★ | ★ | - | - | **5** |
| Goto, 2009 | ★ | ★ | ★ | ★ | ★★ | ★ | ★ | - | **8** |
| Gutiérrez, 2007 | ★ | ★ | ★ | ★ | - | ★ | ★ | - | **6** |
| Huang, 2023 | ★ | ★ | ★ | ★ | ★ | ★ | ★ | - | **7** |
| Iwasaki, 2016 | ★ | ★ | ★ | ★ | ★ | ★ | ★ | - | **7** |
| Koike, 2024 | ★ | ★ | ★ | ★ | ★★ | ★ | - | ★ | **8** |
| Kovács, 2014 | ★ | ★ | ★ | ★ | ★★ | ★ | ★ | ★ | **9** |
| Le, 2012 | ★ | ★ | ★ | - | ★ | ★ | ★ | - | **6** |
| Le, 2014 | ★ | ★ | ★ | - | ★ | ★ | ★ | - | **6** |
| Lee, 2012 | ★ | ★ | ★ | - | ★ | ★ | ★ | ★ | **7** |
| Li, 2022 | ★ | ★ | ★ | ★ | ★★ | ★ | ★ | - | **8** |
| Liu, 2021 | ★ | ★ | ★ | ★ | ★ | ★ | - | - | **6** |
| Liu, 2023 | ★ | ★ | ★ | ★ | - | ★ | ★ | ★ | **7** |
| Ma, 2020 | ★ | ★ | ★ | ★ | ★★ | ★ | ★ | ★ | **9** |
| Mahanta, 2019 | ★ | ★ | ★ | - | - | ★ | ★ | ★ | **6** |
| Manno, 2007 | ★ | ★ | ★ | - | ★★ | ★ | ★ | - | **7** |
| Matsuzaki, 2021 | ★ | ★ | ★ | ★ | ★★ | ★ | ★ | - | **8** |
| Miyabe, 2021 | ★ | ★ | ★ | ★ | - | ★ | ★ | - | **6** |
| Nagai, 2022 | ★ | ★ | ★ | ★ | ★★ | ★ | ★ | - | **8** |
| Neves, 2020 | ★ | ★ | ★ | ★ | ★★ | ★ | ★ | - | **8** |
| Pan, 2018 | ★ | ★ | ★ | ★ | ★★ | ★ | - | - | **7** |
| Rafalska, 2014 | ★ | ★ | ★ | ★ | ★ | ★ | - | - | **6** |
| Rivedal, 2025 | ★ | ★ | ★ | ★ | ★ | ★ | ★ | ★ | **8** |
| Rui, 2022 | ★ | ★ | ★ | ★ | ★ | ★ | ★ | - | **7** |
| Sevillano, 2017 | ★ | ★ | ★ | ★ | ★ | ★ | ★ | - | **7** |
| Shen, 2007 | ★ | ★ | ★ | ★ | - | ★ | ★ | - | **7** |
| Shen, 2008 | ★ | ★ | ★ | ★ | ★ | ★ | ★ | ★ | **8** |
| Shen, 2010 | ★ | ★ | ★ | ★ | ★★ | ★ | ★ | - | **8** |
| Shu, 2017 | ★ | ★ | ★ | - | - | ★ | ★ | ★ | **6** |
| Tan, 2022 | ★ | ★ | ★ | ★ | ★★ | ★ | ★ | ★ | **9** |
| Tanaka, 2015 | ★ | ★ | ★ | - | ★★ | ★ | - | - | **6** |
| Wakai, 2006 | ★ | ★ | ★ | ★ | ★ | ★ | ★ | ★ | **8** |
| Weng, 2022 | ★ | ★ | ★ | ★ | ★ | ★ | ★ | - | **7** |
| Xing, 2024 | ★ | ★ | ★ | ★ | ★ | ★ | ★ | - | **7** |
| Yano, 2024 | ★ | ★ | ★ | ★ | ★★ | ★ | - | ★ | **7** |
| Yasuda, 2024 | ★ | ★ | ★ | - | ★ | ★ | - | - | **5** |
| Yu, 2020 | ★ | ★ | ★ | ★ | ★★ | ★ | - | ★ | **8** |
| Zhang, 2016 | ★ | ★ | ★ | - | - | ★ | ★ | - | **5** |
| Zhang, 2024 | ★ | ★ | ★ | - | - | ★ | - | ★ | **5** |

**Abbreviation:** NOS, Newcastle-Ottawa Scale.
**Note:** Each study was assessed across three domains: selection (maximum 4 stars), comparability (maximum 2 stars), and outcome (maximum 3 stars), for a total possible score of 9 stars. ★ indicates one star awarded; ★★ indicates two stars awarded (comparability domain only). A dash (-) indicates no star awarded. Higher scores indicate higher study quality.

### Table S4. Exclusion reasons for studies identified in the SLR and not included in the meta-analysis

| **Study** | **Hematuria measurements** | **Reason for exc**l**usion** |
| --- | --- | --- |
| Bobart, 2021 | Persistent microhematuria | The study analyzed the rate of decline in eGFR using a GEE model, and did not report a hazard ratio. |
| Huang, 2023 | Persistent microhematuria | Hematuria was defined using a higher threshold than other studies, limiting comparability across studies. |
| Le, 2012 | Persistent microhematuria | Hematuria was analyzed as a continuous rather than as a binary variable (microhematuria yes vs. no). |
| Weng, 2022 | Persistent microhematuria | Hematuria was analyzed as a continuous rather than as a binary variable (microhematuria yes vs. no). |
| Ebbestad, 2022 | Persistent microhematuria | Only p-values were reported without effect size estimates. |
| Matsuzaki, 2021 | Persistent microhematuria | The study analyzed the rate of decline in eGFR using a linear regression model, and did not report a hazard ratio. |
| Yano, 2024 | Persistent microhematuria | Hematuria was analyzed using trajectory-based categories rather than a comparison between patients with and without persistent hematuria. |
| Zhang, 2024 | Initial microhematuria | Odds ratios were reported without corresponding hazard ratios. |
| Nagai, 2022 | Initial microhematuria | The study analyzed the rate of decline in eGFR using a linear regression model, and did not report a hazard ratio. |
| Tan, 2022 | Initial microhematuria | Hematuria was analyzed as a continuous rather than as a binary variable (microhematuria yes vs. no). |
| Iwasaki, 2016 | Initial microhematuria | Hematuria was analyzed as a continuous rather than as a binary variable (microhematuria yes vs. no). |
| Rafalska, 2014 | Initial microhematuria | Odds ratios were reported without corresponding hazard ratios. |
| Shen, 2007 | Initial microhematuria | Odds ratios were reported without corresponding hazard ratios. |
| Shen, 2008 | Initial microhematuria | Odds ratios were reported without corresponding hazard ratios. |
| Shen, 2010 | Initial microhematuria | Odds ratios were reported without corresponding hazard ratios. |
| Neves, 2020 | Initial microhematuria | Odds ratios were reported without corresponding hazard ratios. |
| Liu, 2021 | Initial microhematuria | Odds ratios were reported without corresponding hazard ratios. |
| Liu, 2023 | Initial microhematuria | Hematuria was analyzed as a continuous rather than as a binary variable (microhematuria yes vs. no). |
| Miyabe, 2021 | Initial microhematuria | Hematuria was analyzed as a continuous rather than as a binary variable (microhematuria yes vs. no). |
| Rui, 2022 | Initial microhematuria | Hematuria was analyzed as a continuous rather than as a binary variable (microhematuria yes vs. no). |
| Xing, 2024 | Initial microhematuria | Odds ratios were reported without corresponding hazard ratios. |
| Wakai, 2006 | Initial microhematuria | Rate ratios were reported without corresponding hazard ratios. |
| Espinosa, 2009 | Macrohematuria | Risk difference was reported without corresponding hazard ratios. |
| Chacko, 2005 | Macrohematuria | Odds ratios were reported without corresponding hazard ratios. |
| Gutiérrez, 2007 | Macrohematuria | Odds ratios were reported without corresponding hazard ratios. |
| Shu, 2017 | Macrohematuria | Odds ratios were reported without corresponding hazard ratios. |
| Ebbestad, 2022 | Macrohematuria | Only p-values were reported without effect size estimates. |
| Tanaka, 2015 | Macrohematuria | Outcomes reported were not related to ESKD. |

### Table S5. Adjustment variables reported by included studies evaluating hematuria and kidney outcomes in IgAN

| **Study** | **Adjustment variables** |
| --- | --- |
| **Persistent microhematuria - Multivariable** | |
| Huang, 2023 | Age, male sex, BMI, mean arterial pressure, hemoglobin, albumin, serum creatinine, fibrinogen, uric acid, total cholesterol, HDL cholesterol, LDL cholesterol, triglycerides, urine protein remission, MEST-C score, steroid use, immunosuppressant use, RAS inhibitor use |
| Koike, 2024 | RAS inhibitor use, glucocorticoid use, tonsillectomy, RF-RG, age, sex, mean arterial pressure, eGFR, UPER, uric acid |
| Le, 2012 | Time-averaged mean arterial pressure, time-averaged RBC count, eGFR at biopsy |
| Li, 2022 | Sex, MEST-C score, tonsillectomy, steroid/immunosuppressant use, RAS inhibitor use, age, proteinuria, serum creatinine, mean arterial pressure |
| Ma, 2020 | Age, sex, smoking, crescents, time-averaged proteinuria, time-averaged mean arterial pressure |
| Matsuzaki, 2021 | Age, systolic BP, total protein, baseline eGFR, baseline urinary protein |
| Sevillano, 2017 | Age, renal function, time-averaged proteinuria, tubular atrophy/interstitial fibrosis |
| Weng, 2022 | Hypertension, macroscopic hematuria, BUN, hemoglobin, uric acid, eGFR, 24-hour urinary protein, tubular atrophy/interstitial fibrosis, microscopic hematuria, time-averaged serum albumin |
| Yano, 2024 | At index: sex, age, hypertension, diabetes, eGFR; during follow-up: SGLT2 inhibitor, ACEi/ARB, and immunosuppressant use |
| Yasuda, 2024 | Sex, hypertension, eGFR, T-score |
| Yu, 2020 | Age, sex, log-transformed proteinuria, mean arterial pressure, steroid/immunosuppressive agent use |
| **Persistent microhematuria - Univariable** | |
| Bobart, 2021 | Univariable estimate |
| Ebbestad, 2022 | Univariable estimate |
| **Initial microhematuria - Multivariable** | |
| Goto, 2009 | Male sex, age <30 years, family history of chronic renal failure, family history of chronic glomerulonephritis, systolic BP, urine protein, serum albumin <4.0 g/dL, GFR, histologic grade III/IV at initial biopsy |
| Koike, 2024 | RAS inhibitor use, glucocorticoid use, tonsillectomy, RF-RG, age, sex, mean arterial pressure, eGFR, UPER, uric acid |
| Liu, 2021 | Gender, mean arterial pressure, hematuria, serum creatinine, urea nitrogen, cholesterol, hemoglobin, Lee's classification, and MEST-C score |
| Liu, 2023 | Baseline mean arterial pressure, baseline eGFR, baseline proteinuria, fraction of crescents, immunosuppressive treatment |
| Manno, 2007 | Age, sex, hematuria, hypertension, serum creatinine, urinary protein excretion, 3- and 5-grade histologic classifications |
| Rafalska, 2014 | Time-averaged proteinuria, diastolic BP, upper respiratory tract disorder |
| Rivedal, 2025 | Sex, age at diagnosis, CKD stage at diagnosis, proteinuria at diagnosis, systolic/diastolic BP at diagnosis, RAAS inhibitor use during follow-up, immunosuppression use during follow-up |
| Shen, 2007 | Sex, age, microalbuminuria, mesangial proliferation, mesangial matrix widening, glomerular sclerosis, interstitial cell infiltration, tubular atrophy, interstitial fibrosis |
| Shen, 2010 | Age, sex, proteinuria at biopsy, hypertension at biopsy, GFR at biopsy, prednisone therapy, remission, hypertension during follow-up, glomerular lesions, tubulointerstitial lesions, vascular lesions |
| Tan, 2022 | Age, sex, segmental glomerulosclerosis, tubular atrophy/interstitial fibrosis, proteinuria, eGFR |
| Tanaka, 2015 | Sex, mean arterial pressure, serum creatinine, RAS inhibitor use |
| Wakai, 2006 | Sex, age, year of initial renal biopsy, systolic BP, proteinuria, serum total protein, serum creatinine, histologic grade at initial biopsy |
| **Initial microhematuria - Univariable** | |
| Bednarova, 2023 | Univariable estimate |
| Bobart, 2021 | Univariable estimate |
| Iwasaki, 2016 | Univariable estimate |
| Miyabe, 2021 | Univariable estimate |
| Nagai, 2022 | Univariable estimate |
| Neves, 2020 | Univariable estimate |
| Rui, 2022 | Univariable estimate |
| Shen, 2008 | Univariable estimate |
| Xing, 2024 | Univariable estimate |
| Zhang, 2024 | Univariable estimate |
| **Macrohematuria - Multivariable** | |
| Chacko, 2005 | Creatinine clearance, hypertension, age |
| Kovács, 2014 | Tonsillectomy, age, sex, BP, eGFR, proteinuria, ACEi/ARB use, statin use, steroid use |
| Le, 2014 | eGFR, proteinuria, hypertension at biopsy |
| Lee, 2012 | Age, sex, edema/gross hematuria, diabetes, hypertension, cancer, GFR, anemia, albumin, systolic BP, diastolic BP, proteinuria, segmental sclerosis, statin treatment history, RAS-blockade treatment history |
| Mahanta, 2019 | Hypertension, proteinuria, crescents, glomerulosclerosis >=50%, IFTA >=50%, herbal medicines/toxins |
| Pan, 2018 | Sex, eGFR, 24-hour urinary protein, serum albumin, hemoglobin, fibrinogen, systolic/diastolic BP, ARB therapy, glucocorticoid therapy, segmental necrosis, Oxford M/E/T/C scores |
| Shu, 2017 | Mesangial hypercellularity, endocapillary hypercellularity, segmental glomerulosclerosis, tubular atrophy/interstitial fibrosis, eGFR at biopsy, sex, age, uric acid, hemoglobin, albumin, total cholesterol, 24-hour urine protein, hypertension, time-averaged uric acid, time-averaged hemoglobin, time-averaged albumin, time-averaged total cholesterol |
| Weng, 2022 | Hypertension, BUN, hemoglobin, uric acid, eGFR, 24-hour urinary protein, tubular atrophy/interstitial fibrosis, microscopic hematuria, time-averaged hematuria, time-averaged serum albumin |
| **Macrohematuria - Univariable** | |
| Ebbestad, 2022 | Univariable estimate |
| Espinosa, 2009 | Univariable estimate |
| Goto, 2009 | Univariable estimate |
| Gutiérrez, 2007 | Univariable estimate |
| Tanaka, 2015 | Univariable estimate |
| Zhang, 2016 | Univariable estimate |

**Abbreviations:** ACEi, angiotensin-converting enzyme inhibitor; ARB, angiotensin receptor blocker; BMI, body mass index; BP, blood pressure; BUN, blood urea nitrogen; CKD, chronic kidney disease; eGFR, estimated glomerular filtration rate; GFR, glomerular filtration rate; HDL, high-density lipoprotein; IFTA, interstitial fibrosis and tubular atrophy; IgAN, immunoglobulin A nephropathy; LDL, low-density lipoprotein; MEST-C, mesangial hypercellularity, endocapillary hypercellularity, segmental glomerulosclerosis, tubular atrophy/interstitial fibrosis, and crescents; N/A, not applicable; NR, not reported; RAAS/RAS, renin-angiotensin-aldosterone system / renin-angiotensin system; RBC, red blood cell; RF-RG, risk factor and renal grade; SGLT2, sodium-glucose cotransporter-2; UPER, urinary protein excretion rate.
